# A patient with AL amyloidosis presenting with refractory tuberculosis, chest tightness and hypotension: case report

**DOI:** 10.1186/s12890-024-03127-1

**Published:** 2024-07-02

**Authors:** Jun Yang, Mohamed Fahim Fathima Farhath, Huohuan Tian, Linhui Yang, Dan Liu

**Affiliations:** 1 Department of Pulmonary and Critical Care Medicine, West China Hospital, Sichuan University, Chengdu, Sichuan China; 2 Department of Pulmonary and Critical Care Medicine, Langzhong People’s Hospital, Langzhong, Sichuan China; 3 State Key Laboratory of Respiratory Health and Multimorbidity, West China Hospital, Sichuan University, Chengdu, Sichuan China

**Keywords:** Light chain amyloidosis, Case report

## Abstract

**Introduction:**

Immunoglobulin light chain (AL) amyloidosis presents a clinical spectrum characterized by diverse manifestations and involvement of multiple organs, posing a significant diagnostic challenge for physicians.

**Methods and results:**

We present a case of a patient admitted to our hospital due to recurrent cough and sputum, which was initially diagnosed as refractory tuberculosis. Throughout his hospitalization, the patient experienced distressing symptoms, including uncontrollable chest tightness, hypotension, and fever. Noteworthy observations included a persistent elevation in cardiac biomarkers, indicative of cardiac damage. Bronchoalveolar lavage revealed the presence of various pathogenic microorganisms, while bone marrow flow cytometry demonstrated the existence of clonal plasma cells. Additionally, the urine free light chain assay detected the presence of M protein, and the positive congo red staining of the abdominal wall fat biopsy confirmed amyloid deposition in the tissues. Taking into account the patient’s clinical presentation and the examination findings, we reached a conclusive diagnosis of immunoglobulin light chain (AL) amyloidosis.

**Conclusion:**

This case serves as a reminder for physicians to consider rare diseases like AL amyloidosis when patients present with symptoms involving multiple organ systems such as heart, lung and kidney that are unresponsive to conventional treatment options.

## Introduction

Immunoglobulin light chain (AL) amyloidosis presents a clinical spectrum characterized by diverse manifestations and involvement of multiple organs, posing a significant diagnostic challenge for physicians. AL amyloidosis represents a severe manifestation of systemic amyloidosis, characterized by the clonal expansion of CD38 + plasma cells. This clonal expansion leads to an excessive production of immunoglobulin light chains, which subsequently undergo misfolding, forming amyloid fibrils. These misfolded amyloid fibrils deposit in multiple tissues and organs, predominantly affecting the heart and kidneys, leading to significant organ damage [1, 2]. AL amyloidosis is an exceptionally rare disease. A comprehensive study conducted in Minnesota revealed that the incidence of this condition is remarkably low, with only 3–5 individuals diagnosed per one million people [3]. AL amyloidosis is commonly seen in elderly patients with equal proportion of affected males and females [4, 5]. AL amyloidosis has a poor long-term prognosis, it’s highly heterogenous and one third of patients diagnosed with the disease die within 6 months of the diagnosis [6].

In 2022, the National Comprehensive Cancer Network (NCCN) published reviews on the diagnosis and risk assessment of AL amyloidosis [7]. It mentioned that the first step in diagnosing AL amyloidosis is to identify the presence of monoclonal proteins. This should be done through serum protein electrophoresis and immunofixation, 24-hour urine protein collection for electrophoresis/immunofixation, and serum free light chain measurement. Once AL amyloidosis is suspected, a biopsy showing Congo red-positive amorphous deposits with apple-green birefringence under polarized light microscopy is required. Additionally, a bone marrow biopsy is necessary to evaluate for underlying B-cell disorders. In the Chinese Medical Journal, scholars proposed the following diagnostic criteria for AL amyloidosis [8]: (1) Congo red-positive staining of biopsy tissue; (2) Identification of light chain deposits in affected tissue using immunohistochemistry, immunofluorescence, or laser microdissection combined with mass spectrometry; (3) Exclusion of multiple myeloma, Waldenström’s macroglobulinemia, and other indolent lymphomas.

Here, we present a rare case of a patient presenting with refractory tuberculosis, chest tightness and hypotension who was finally diagnosed with AL amyloidosis.

## Case presentation

The patient, a 49-year-old male, was admitted to the hospital with recurrent cough and sputum for 8 years with exacerbation for 20 days. Eight years prior to this admission, the patient had repeated episodes of cough and sputum, and was diagnosed with “pulmonary tuberculosis, bronchial tuberculosis, and tuberculous pleurisy” at other hospitals. The patient intermittently underwent repeated anti-tuberculosis treatment during this period. Twenty days prior to admission, the patient again experienced the aforementioned symptoms. Further examinations at local hospital indicated: tests for tuberculosis such as T-cell gamma interferon release test, sputum smear and sputum culture were all negative. Upon receiving anti-infection treatment at local hospital, the patient reported aggravation of the cough and shortness of breath. The patient was then transferred to our emergency department for further evaluation of his condition.

**Fig. 1:**
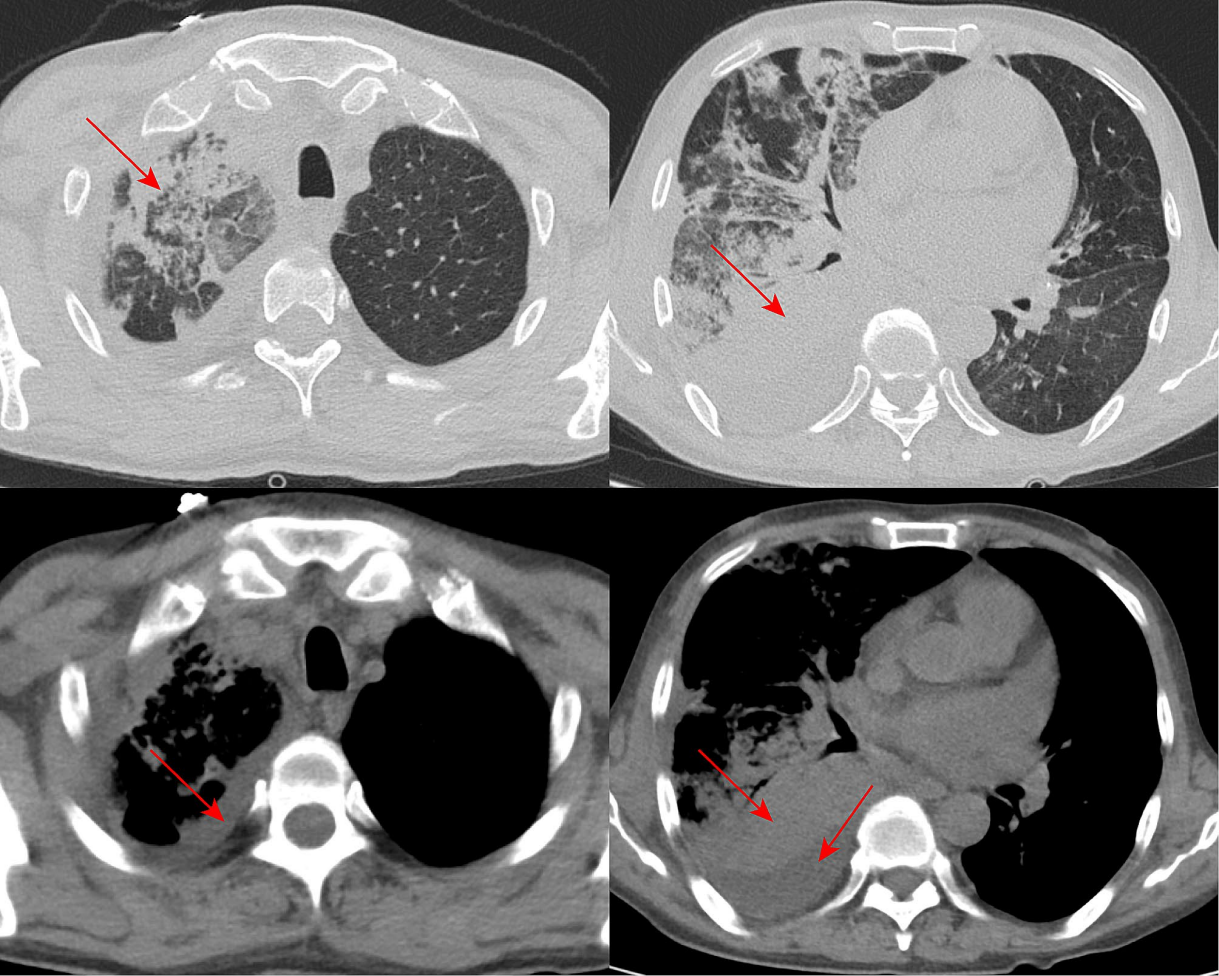
CT chest scan of the patient on admission to the emergency room

Upon arrival at the emergency department, the patient completed a chest CT, which suggested scattered solid, atelectasis in both lungs, with bronchial inflation signs visible in some of the lesions, predominantly on the right side, in addition to a small to moderate amount of pleural effusion visible on the right side and compression of adjacent lung tissue (Fig. 1). Bedsides, ultrasound suggested that the patient had left ventricular hypertrophy, left atrial enlargement, and slightly reduced left ventricular systolic function measurements (Ejection fraction-EF: 45%). The blood test results at the emergency department revealed a noteworthy decrease in the absolute lymphocyte values (0.49 × 10^9, reference range 1.1–3.2 × 10^9), a reduced level of globulin at 19.5 g/L (reference range 20–40 g/L), elevated troponin T147.1 ng/L (reference range 1–14 ng/L), and significantly increased levels of N-terminal pro-B-type natriuretic peptide (NT-pro BNP) exceeding 35,000 ng/L (reference range less than 88 ng/L). In the pathogen examination, the patient’s sputum revealed Candida albicans and a significant number of Gram-negative bacilli. Upon admission to the emergency department, the patient was treated with meropenem for infection as well as other symptomatic treatments, including diuretics.

**Fig. 2:**
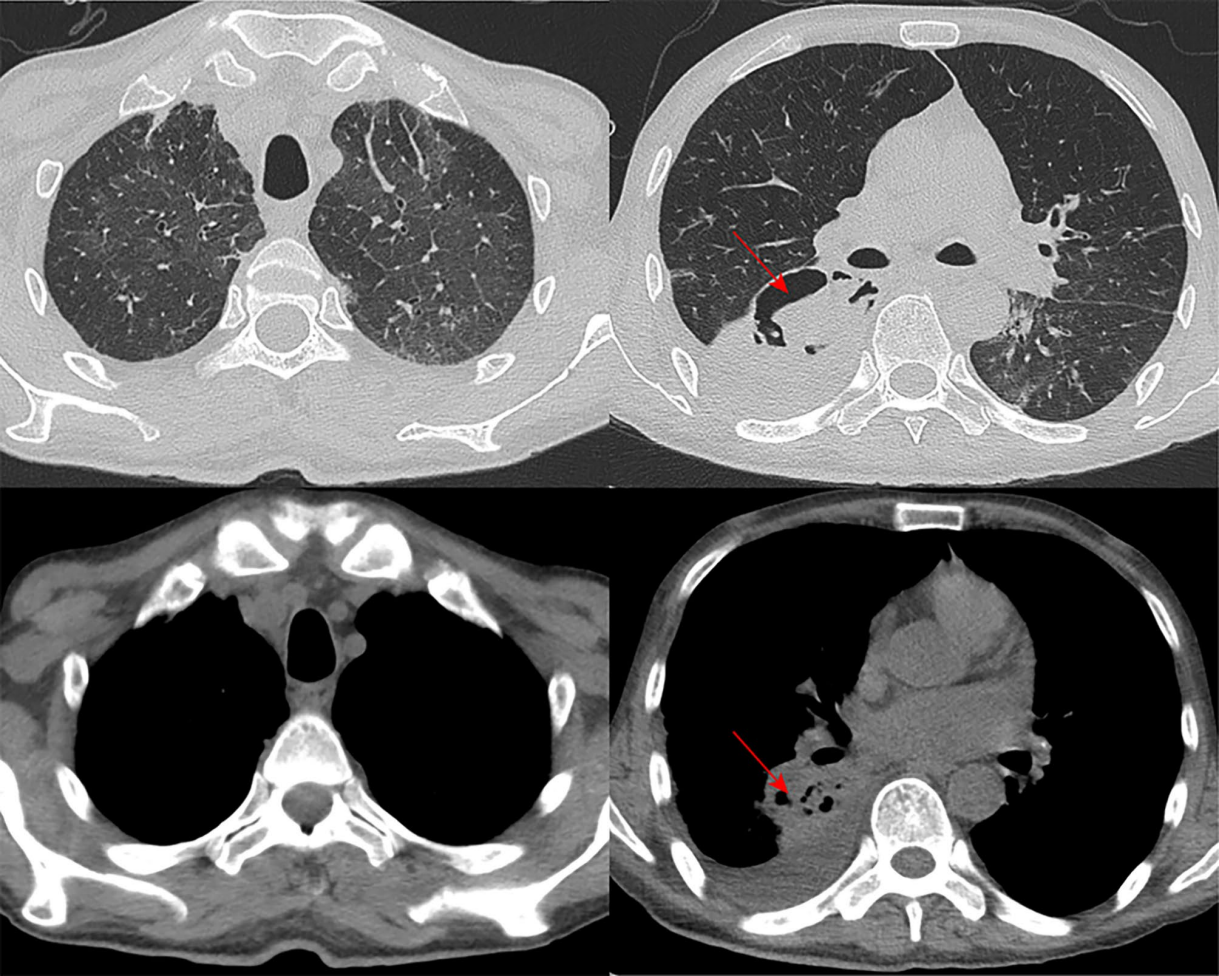
A follow-up chest CT after the patient was admitted to the hospital for treatment

Upon transfer to the respiratory unit, the patient continued to exhibit significant symptoms of cough, shortness of breath, chest tightness, persistent hypotension, and intermittent fever, with the highest temperature reaching 39.8 °C during febrile episodes. The auxiliary examination results upon admission showed the following: the gram stain of the patient’s sputum smear indicated the presence of small number of gram-stain negative bacilli, gram positive streptococci, and yeast like bacteria. Sputum culture showed the growth of candida albicans. A follow-up blood test revealed a markedly reduced serum immunoglobulin level (0.49 g/L) and a CD4 + T cell count of only 240 cells/µL (reference range: 471–1220 cells/µL). Given the patient’s recurrent fever despite the use of meropenem, the detection of Gram-positive bacteria in the sputum, and the patient’s compromised immune function, vancomycin was added to the meropenem regimen for anti-infective therapy. During this period, due to renal function impairment in the patient, we modified the antimicrobial regimen to moxifloxacin in combination with linezolid for bacterial treatment. In addition, the myocardial markers were elevated compared to the initial results (troponin T 204.7ng/L). Given the patient’s chest tightness and persistently elevated troponin levels, we could not rule out the possibility of acute coronary syndrome. Consequently, a coronary CT scan was performed, revealing no significant abnormalities in the coronary vessels. Unfortunately, the patient was unable to undergo a cardiac MRI as he was unable to lie down for an extended period. During this period, a follow-up chest CT scan revealed irregular cavity formation within the consolidation of the right lower lobe of the patient’s lung (Fig. 2). In conjunction with the previous sputum microbiology results, we considered the possibility of fungal infection in the patient and initiated antifungal treatment with caspofungin.

**Fig. 3:**
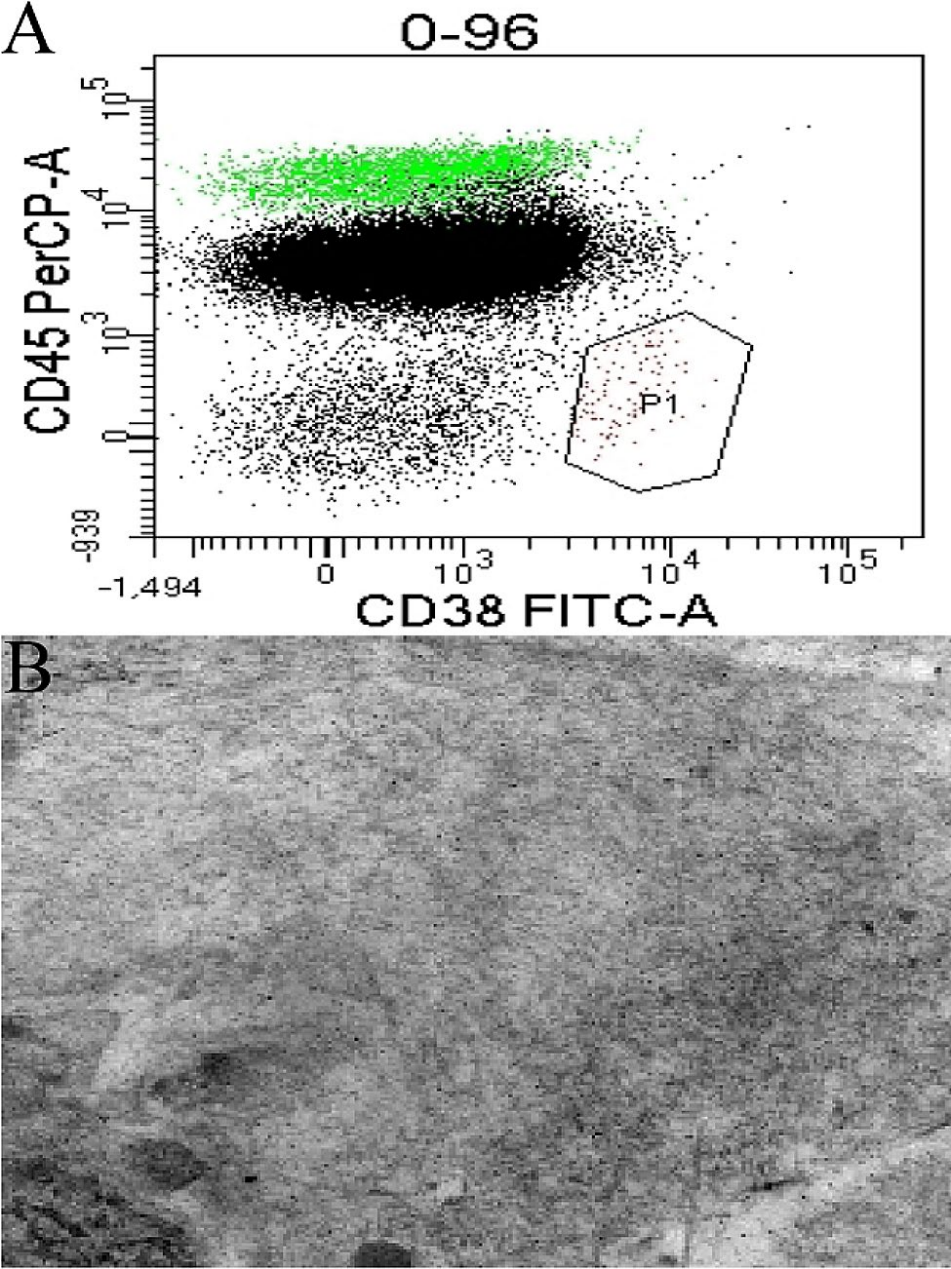
(**A**) Results of the patient’s bone marrow flow cytometry analysis. (**B**) Electron microscopic presentation of the patient’s abdominal wall fat biopsy

Through multidisciplinary consultations, we determined that the patient presents with a lung infection concomitant with immunoglobulin deficiency, warranting appropriate supplementation. Besides, concurrent screening for hematological disorders and further refinement of pulmonary pathogen investigation were necessary. Moreover, while coronary arteries show no significant abnormalities, the sustained elevation of troponin levels could not exclude the possibility of myocardial amyloidosis. Therefore, we conducted concurrent antimicrobial treatment for the patient while refining the corresponding screenings. The results are as follows: the Patient’s serum immunofixation electrophoresis detected no M-protein. And the urine protein measurement indicated a mild elevation in urinary protein levels (1 g/L). Following assessment and alleviation of the patient’s heart failure and fever, we conducted a fiberoptic bronchoscopy examination. Bronchoscopy examination revealed abundant purulent secretions in both bronchi. Bacterial culture of bronchoalveolar lavage fluid (BALF) indicated the presence of Acinetobacter baumannii, while fungal culture suggested the presence of Candida albicans, with negative results for tuberculosis-associated examination. Moreover, metagenomic next-generation sequencing (mNGS) of the BALF indicated the presence of Pseudomonas aeruginosa (sequence count: 227), Candida species (sequence count: 5), Aspergillus fumigatus (sequence count: 3), Human alphaherpesvirus 1 (sequence count: 662), Human adenovirus D species (sequence count: 15), Circovirus (sequence count: 6), and Human metapneumovirus (sequence count: 9181). The patient’s bone marrow flow results were obtained 28 days after admission and suggested the presence of a few clonal plasma cells detected by FCM analysis (Fig. 3A). However, due to family financial reasons, the patient’s family decided to leave the hospital.

One week after discharge, a subcutaneous fat biopsy of the patient’s abdominal wall revealed a significant number of amyloid microfilament deposits in the interstitial space of adipocytes and next to the wall of small vessels (Fig. 3B), with positive Congo red staining. Additionally, λ-type M protein was detected in the patient’s urine. Finally, the patient was diagnosed with λ-type systemic light chain amyloidosis which was a result of AL-type amyloidosis secondary to clonal plasmacytosis. One month after the patient was discharged, we conducted a follow-up and found that the patient had passed away approximately two weeks after discharge.

## Discussion

The clinical manifestations of AL amyloidosis exhibit remarkable diversity and involve multiple tissues and organs. Current literature reports that up to 70% of patients with AL amyloidosis experience cardiac involvement [9]. In this case, cardiac symptoms were the most prominent. The patient initially presented with recurrent chest tightness, and serum tests for myocardial markers indicated progressively increasing levels of troponin and NT-proBNP. Despite the absence of significant vascular lesions on coronary CT, the patient subsequently exhibited signs of heart failure, including hypotension and dyspnea. In patients with AL amyloidosis, cardiac involvement is primarily assessed through ECG, NT-proBNP, troponin, echocardiography, and cardiac MRI [10]. According to the 2023 NCCN guidelines, cardiac involvement can be determined by excluding other cardiac diseases if the ventricular wall thickness exceeds 12 mm and NT-proBNP elevation is not due to renal impairment or atrial fibrillation [11]. In AL amyloidosis, renal involvement occurs in up to 80% of patients [12]. In the reported case, the patient’s renal involvement was not pronounced, presenting only as proteinuria and mildly elevated renal function. However, based on the patient’s urine protein and urine light chain electrophoresis results, we considered that the kidneys were also affected by amyloidosis. Renal amyloidosis must be carefully differentiated from nephrotic syndrome, as both can present with proteinuria, edema, and renal insufficiency. When proteinuria is accompanied by systemic symptoms (such as heart failure and gastrointestinal symptoms), renal amyloidosis should be highly suspected. However, a definitive diagnosis requires confirmation through a renal biopsy [12]. In addition to cardiac and renal involvement, our reported case also revealed amyloid protein deposits in the abdominal fat tissue. Literature has indicated that abdominal fat pad biopsy has a high sensitivity for diagnosing AL amyloidosis [13]. Thus, abdominal fat pad biopsy offers a less invasive diagnostic method. In the reported case, although no evidence of tuberculosis was detected in the sputum and bronchoalveolar lavage fluid during this hospitalization, ruling out tuberculosis as the cause of the respiratory symptoms, fungi and various bacteria were identified in these samples. Furthermore, follow-up CT scans after anti-infective treatment showed lesion resolution. Therefore, we believe the patient’s pulmonary changes were related to fungal and bacterial infections. Due to the lack of a comprehensive lung biopsy and Congo red staining, we cannot determine whether pulmonary amyloidosis is also present at the same time.

The non-specific clinical presentation of AL amyloidosis, as evident from the reported case, contributes to delayed diagnosis and poor prognosis, which leads to progressive disability and mortality [2]. Early diagnosis is vital to prevent irreversible organ damage. A comprehensive diagnosis of AL amyloidosis entails confirming amyloid deposition, determining the fiber type, evaluating disease severity, and assessing the extent of organ involvement [14]. We present a case of a patient who initially presented with respiratory symptoms, specifically recurrent cough and sputum, which revealed an underlying incurable tuberculosis infection. During hospitalization, a bronchoalveolar lavage test detected a variety of pathogenic microorganisms, indicating a compromised immune system. This raised suspicion of an immune system abnormality in the patient, leading to further investigation. Throughout the hospitalization, the patient continued to exhibit significant symptoms of cough, shortness of breath, chest tightness, persistent hypotension, and intermittent fever. Although a coronary CT scan showed no significant abnormalities, the patient exhibited a persistent elevation in cardiac biomarkers, suggesting myocardial damage. Subsequent cardiac ultrasound revealed impaired cardiac diastolic function and septal thickening, prompting consideration of myocardial amyloidosis. Given the patient’s poor cardiac function and general condition, which precluded prolonged cardiac MRI and lung biopsy, we chose to perform a biopsy on the abdominal fat, where obtaining a sample is relatively easier. Concurrently, we conducted urine immunofixation electrophoresis, which detected the presence of λ-type M protein. These measures ultimately led to the diagnosis of AL amyloidosis.

This case highlights the diagnostic challenges encountered, as the initial presentation primarily involved respiratory symptoms, leading to a focus on anti-pulmonary infection treatment. AL amyloidosis commonly affects the heart and kidneys, which were not initially considered. The progression of cardiac symptoms and the patient’s deteriorating condition eventually led to suspicion and subsequent diagnosis of AL amyloidosis. At last, we consider that AL amyloidosis may have involved the heart, kidneys and subcutaneous fat of the patient. This case serves as a reminder for physicians to consider rare diseases like AL amyloidosis when patients present with symptoms involving multiple organ systems that are unresponsive to conventional treatment options.

## Data Availability

All data generated or analysed during this study are included in this published article.
